# High frequency of tumor cells with nuclear Egr-1 protein expression in human bladder cancer is associated with disease progression

**DOI:** 10.1186/1471-2407-9-385

**Published:** 2009-10-30

**Authors:** Frederikke Lihme Egerod, Annette Bartels, Niels Fristrup, Michael Borre, Torben F Ørntoft, Martin B Oleksiewicz, Nils Brünner, Lars Dyrskjøt

**Affiliations:** 1 Cell and Antibody Analysis, Novo Nordisk A/S, Novo Nordisk Park, DK-2760 Maaloev, Denmark; 2 Section of Pathobiology, Department of Veterinary Disease Biology, Dyrlægevej 88, DK-1870 Frederiksberg C, Denmark; 3 Molecular Diagnostic Laboratory, Department of Molecular Medicine, Aarhus University Hospital, Skejby, Science Center Skejby, Brendstrupgaardsvej 21, DK-8200 Aarhus N, Denmark; 4 Department of Urology, Aarhus University Hospital, Skejby, DK-8200 Aarhus N, Denmark; 5 Molecular Microbiology, Intercell AG, Campus Vienna Biocenter 3, 1030 Vienna, Austria

## Abstract

**Background:**

Egr-1 (early growth response-1 transcription factor) has been proposed to be involved in invasion and metastasis processes of human bladder cancer, but Egr-1 protein expression levels in human bladder cancer have not been investigated. In the present study we investigated the expression levels of Egr-1 protein in early stages of human bladder cancer and correlated it to later progression.

**Methods:**

Expression of Egr-1 protein in human bladder cancer was examined by immunohistochemistry, on a tissue microarray constructed from tumors from 289 patients with non-muscle invasive urothelial bladder cancer.

**Results:**

The frequency of tumor cells with nuclear Egr-1 immunolabelling correlated to bladder cancer stage, grade and to later progression to muscle-invasive bladder cancer (T2-4). Stage T1 tumors exhibited significantly higher frequencies of tumor cells with nuclear Egr-1 immunolabelling than Ta tumors (P = 0.001). Furthermore, Kaplan-Meier survival analysis showed that a high frequency of tumor cells with nuclear Egr-1 immunolabelling was significantly associated with a higher risk of progression to stage T2-4 (log-rank test, P = 0.035). Tumor cells with nuclear Egr-1 immunolabelling were found to localize at the tumor front in some of the tumor biopsies.

**Conclusion:**

The results from this study support a potential involvement of Egr-1 in the progression from non-muscle invasive bladder cancers to muscle invasive bladder cancer.

## Background

Human bladder cancer is the forth most common malignancy in men, and the tenth most common in women [1]. The majority of malignant bladder tumors are urothelial cell carcinomas evolved from the epithelial lining of the bladder wall (urothelium). These tumors can be further divided into papillary, solid and carcinoma *in situ *(CIS) lesions. Papillary tumors are the most common type, they tend to grow slowly. Solid tumors are less frequent and more aggressive and infiltrate the muscular layer of the bladder wall. CIS is a lesion involving only the inner lining of the bladder. Bladder tumors are classified according to the depth of invasion: non-invasive Ta, and lamina-propria invasive but not muscle-invasive T1 tumors, and muscle-invasive T2-4 tumors. More than 60% of the Ta tumors recur, which makes this tumor type mainly responsible for the high prevalence rate. About 40% of the patients experience multiple recurrences, which has a significant impact on the quality of life.

Studies in urothelial carcinoma cells have demonstrated different gene expression profiles in non-muscle invasive and muscle invasive tumors and molecular classifiers of urothelial cell carcinoma outcome have been identified [2]. Furthermore, different biomarker proteins have been investigated to diagnose and prognosticate bladder cancers [3-6]. However, more information about different molecular subtypes and molecular pathways of early stage bladder tumors might ultimately facilitate prediction of disease outcome and treatment response.

Egr-1 (early growth response factor 1, Zif268, NGFI-A, TIS8, Krox-24) has been proposed to be involved in the invasion and metastasis processes of human bladder cancer [7,8]. In both bladder and prostate cancer Egr-1 is shown to regulate the expression of heparanase and hyaluronidase, which are implicated in the metastatic spread of cancer cells [8-10]. Egr-1 is a zinc finger transcription factor involved in cellular responses to mitogens, growth factors and stress stimuli. Egr-1 has also been proposed to be an early biomarker in rat urinary bladder cancer induced by dual-acting PPAR agonists [11]. Egr-1 is induced by epidermal growth factor (EGF) and has been shown to correlate to EGF receptor (EGFr) levels in bladder tumors [7]. The EGFr is a recognized bladder tumor marker and high levels of EGFr are associated with non-papillary, high-grade invasive tumors [12].

To our knowledge only the Egr-1 mRNA but not the protein expression levels in human bladder cancer have been previously investigated [8]. In the present study we investigated the expression levels of Egr-1 protein in early stages of human bladder cancer and correlated it to later progression.

The REMARK recommendations were followed when feasible [13].

## Methods

### Patient information

Bladder tumor biopsies were obtained with informed written consent from the patients. The study was approved by The Scientific Ethical Committee of the County of Aarhus (1994/2920). All tumors selected for this study were primary urothelial tumors, stage Ta or T1. The patient material was collected from 1979 to 2007. Median follow-up time was 74 months (range 1-232 months). The patient follow-up was from the first diagnostic resection to the most recent cystoscopy. In case of death before progression or before a five year follow-up without progression, the patients were excluded from the study. The event of progression to muscle invasive bladder cancer was verified by pathological examination. Patients who underwent cystectomy before pathological evidence of progression were excluded from the study. Two patients received chemotherapy during the course of the disease and 56 patients received bacillus Calmette-Guérin (BCG) immunotherapy. 101 patients had concomitant CIS during follow-up and of these 38 patients received BCG immunotherapy.

### Biological material

At the Institute of Pathology, Aarhus University Hospital, Denmark, formalin fixed paraffin embedded urothelial tumor blocks were selected for this study. A total of 289 patients with primary, non-muscle invasive urothelial tumors (182 pTa, 101 pT1, 6 CIS) fulfilled the inclusion criteria for the study; 118 tumors progressed to muscle invasive bladder cancer (pT2-T4) during follow-up. The remaining 171 patients were followed for at least five years and none showed progression to muscle invasive bladder cancer.

The original Haematoxylin-Eosin (HE) stained sections were reviewed by an experienced uropathologist who re-evaluated the stage and grade of each tumor blinded to the original diagnosis. Grading was performed according to WHO 2004 classification. The original slides for tumors diagnosed before 1997 were not available, and consequently new HE stained sections were made and evaluated by the same uropathologist, who also identified the tumor regions of each paraffin embedded bladder cancer for the following tissue microarray construction. Tissue samples of normal urothelium were taken from persons with no history of bladder tumors, but who suffered from unknown bladder pathologies that indicated bladder biopsy.

Sections of paraffin embedded human prostate cancer specimens (n = 3) (a kind gift from the Pathology Department, Rigshospitalet, Denmark) were used as positive controls for Egr-1 immunolabelling, as Egr-1 is found overexpressed in the majority of human prostate cancers [14-17].

### Tissue microarray construction

One biopsy (0.6 mm cores) from every tumor was taken from the area marked by the uropathologist and placed in the recipient paraffin block using a custom-made precision instrument (manual tissue microarrayer 1, Beecher Instruments Inc., Sun Prairie, WI, USA). This was done according to the method developed by Kononen [18].

### Antibodies

Egr-1 rabbit monoclonal antibody (Cell Signaling Technology, California, USA, catalogue number 4153) was used at 1:750 dilution.

Biotinylated secondary antibody, polyclonal goat anti-rabbit (Dako A/S, Denmark, catalogue number E0432) was used at 1:2000 dilution.

### Immunohistochemistry

The TMA was dewaxed in xylene and rehydrated through a graded ethanol series. Antigen retrieval was performed by boiling for 10 minutes in 10 mM citric acid buffer, pH 6.0 in a microwave oven. Endogeneous peroxidase activity was blocked by incubation with 3% (v/v) hydrogen peroxide for 10 minutes. Endogenous binding sites were blocked using an avidin/biotin blocking kit (Vector laboratories Inc., California, USA, catalogue number SP-2001) according to the manufacturer's protocol. The TMA was incubated with avidin for 10 minutes and washed twice in TBST wash buffer (0.05 M Tris-HCl pH 7.6, 0.3 M NaCl and 0.1% Tween 20) followed by biotin incubation for 10 minutes.

Immunohistochemistry for Egr-1 was done using a tyramide-based catalyzed signal amplification kit, according to the manufacturer's recommendations (Dako A/S, Denmark, catalogue number K1500). All incubations and washes were done at ambient temperature. The TMA was washed twice in TBST and blocked in protein block for 5 minutes, followed by 15 minutes incubation with anti-Egr-1 antibody in antibody diluent (Dako A/S, Denmark, catalogue number S3022). The TMA were washed 3 times in TBST and underwent 5 sequential incubations with 3× washes in TBST between each step: 15 minutes with biotinylated goat anti-rabbit IgG (diluted in TBST); 15 minutes with streptavidin-biotin complex; 15 minutes with amplification reagent; 15 minutes with streptavidin-peroxidase; and 5 minutes with substrate chromagen solution. Finally, the TMA were rinsed in water, counterstained with Mayer's haematoxylin, dehydrated through ethanol into xylene and mounted in DPX (Fisher Scientific, Loughborough, UK, catalogue number D/5319/05).

**Figure 1:**
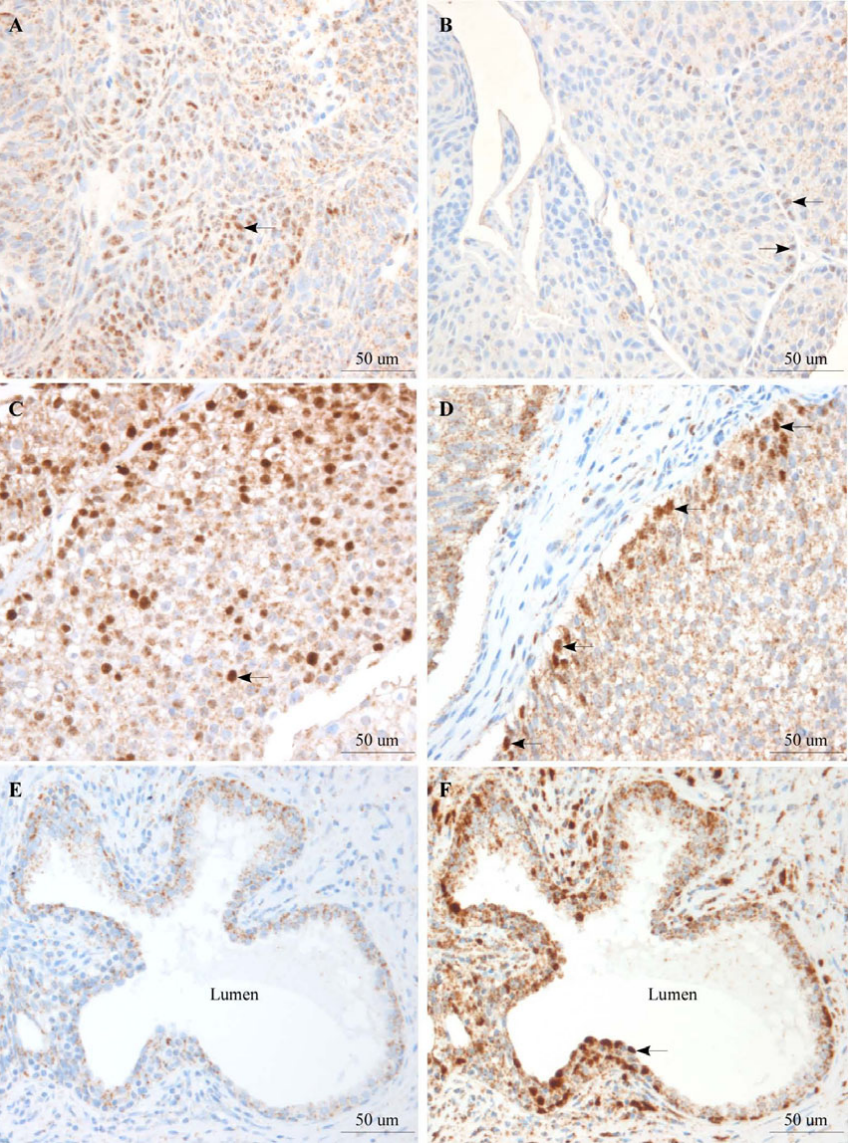
**Egr-1 expression in human bladder cancer and prostate cancer tissue**. Expression of Egr-1 was examined by immunohistochemistry in human bladder cancer tissue cores arranged on a tissue microarray (A-D), and in prostate cancer tissue (E, F). E, negative control with the primary Egr-1 antibody omitted. Arrow, nuclear Egr-1 immunolabelling.

The specificity of the rabbit monoclonal antibody used in this study has been evaluated by the use of another polyclonal anti-Egr-1 antibody (SC-189, Santa Cruz Biotechnology), and full agreement between the two was found [11,19] and Egerod et al., 2009, submitted. Furthermore, the observed nuclear localization of Egr-1 was in agreement with that observed by others [20]. Finally, a majority of human prostate cancer cells exhibited strong nuclear Egr-1 immunolabelling, in agreement with the known high expression of Egr-1 in human and mouse prostate cancers [14-17]. No nuclear Egr-1 immunolabelling was found in the negative prostate cancer control where the primary antibody was omitted (figure 1 E-F). The positive and negative control followed the same immunostaining procedure as the TMA, with the exception for the negative control where the primary anti-Egr-1 antibody was omitted.

### Semiquantitation of Egr-1 immunolabelling

Egr-1 immunolabelling was scored (semiquantitatively) by two persons using a double headed microscope, according to predefined scoring criteria (see below). Scoring was blinded to the course of the disease.

Egr-1 nuclear immunolabelling was scored only in tumor cells (based on purely morphological criteria, using only haematoxylin staining, and leaving out small lymphocyte-like cells). Specimens were scored as "0" when no nuclear Egr-1 labelling was present, "1" when less than 33% of tumor cell nuclei exhibited Egr-1 immunolabelling, "2" when between 33% and 67% of tumor cell nuclei exhibited nuclear Egr-1 immunolabelling, and "3" when over 67% of the tumor cells exhibited nuclear immunolabelling. The intensity of the nuclear immunolabelling was likewise scored visually in categories, "1" for weak nuclear staining intensity, "2" for moderate nuclear staining intensity and "3" for strong nuclear staining intensity.

The reproducibility of the Egr-1 immunolabelling was evaluated by a second score of approximately 30 biopsies randomly chosen from a second TMA stained on a different day. The scores between the two TMAs were identical.

### Statistics

Kruskal-Wallis and Mann Whitney tests were done using GraphPad Prism 5 software (GraphPad Software, Inc., CA, USA). Stata 10.0 statistical analysis software (Stata Corporation, College Station, TX, USA) was used for calculation of Log-rank tests for equality of survival function, Kaplan-Meier survival plots, and univariate and multivariate Cox regression analysis. Variables with a P value < 0.01 in univariate analysis were included in multivariate analysis to identify variables with independent significance. The assumptions of proportional hazards were verified.

## Results

Of the 289 tissue cores on the TMA, some tissue cores were missing, likely due to loss during sectioning or antigen retrieval. In total 244 tissue cores (158 Ta tumors, 84 T1 tumors and 2 CIS lesions) were scored for Egr-1 protein expression (table 1). The 2 CIS lesions represented on the TMA were too few for statistical analysis. Only 5 tissue cores were scored as category 3 (high Egr-1 score), and consequently, in order to perform statistical analysis the two Egr-1 scores of 2 and 3, were combined in one group.

**Table 1:** Patient and tumor characteristics

Characteristics	**Number**^a^	Number Egr-1 positive biopsies	Percent Egr-1 positive biopsies	P
**All patients**	244			

**Sex**				

Male	193	109	56.2	0.882^d^

Women	51	28	54.9	

**Tumor size**				

< 3 cm	153	81	52.9	0.282^d^

> 3 cm	63	40	63.5	

**Tumor type**				

Papillary	213	118	55.4	0.033^e^

Solid	16	7	43.8	

Mixed	13	12	92.3	

**Tumor stage**				

Ta	158	79	50.0	0.001^d^

T1	84	58	69.0	

CIS^b^	2	0		

**Histological grade**				

PUNLMP^c ^+ Low grade	158	78	49.4	< 0.001^d^

High grade	83	58	69.9	

^a^Of the 289 biopsies on the TMA, analysis was only possible of 244 biopsies

^b^Excluded from analysis (too few represented)

^c^Papillary urothelial neoplasm of low malignant potential

^d^Mann Whitney test

^e^Kruskal-Wallis test.

### Egr-1 expression levels in relation to patient and tumor characteristics

Egr-1 expression in 244 tumors was compared to clinical and histopathological characteristics. The frequency and number of Egr-1 positive tumors are listed in table 1. We found that Egr-1 expression was correlated to tumor stage, stage T1 tumors exhibited significantly higher levels of Egr-1 positive tumor cells than Ta tumors (P = 0.001, Mann Whitney test). Egr-1 expression levels were also significantly correlated to the tumor growth patterns. The highest Egr-1 expression was found in tumors showing both solid and papillary growth, and lowest expression was observed in solid tumors (P = 0.033, Kruskal-Walis test). Finally, we found that Egr-1 expression was significantly correlated to tumor grade. The high grade tumors showed significantly higher levels of Egr-1 positive tumor cells compared to low grade tumors or PUNLMP (papillary urothelial neoplasm of low malignant potential) (P < 0.001, Kruskal-Walis test). There were no significant differences in Egr-1 expression when comparing sex or tumor size.

### Correlation between Egr-1 expression levels and progression free survival

**Figure 2:**
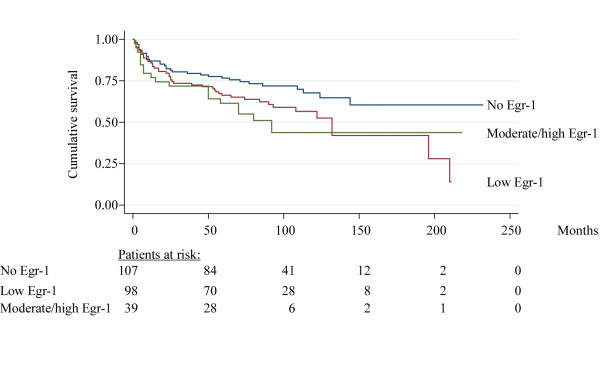
**Kaplan-Meier plot**. Kaplan-Meier survival curve illustrating correlation between Egr-1 score (frequency of Egr-1 positive cancer cell nuclei in tumor mass) and progression to stage T2-T4 bladder cancer. Information from 244 patients was included. P = 0.035, log rank test. Total number of events were 96, with 33 events in Egr-1 negative, 44 events in low Egr-1 and 19 invents in moderate/high Egr-1.

Kaplan-Meier survival statistics showed that tumors with high percentages of Egr-1 positive tumor nuclei had a significantly higher risk of progression to stage T2-4 (log-rank test, P = 0.035, figure 2). The survival curves presented in figure 2 suggest that patients with lack of Egr-1 protein in the cancer cells constitute a seperat subpopulation with a more favourable prognosis than patients with either low, moderate or high Egr-1 protein expression. Indeed separating the patients into only two groups (no Egr-1 versus Egr-1 protein) resulted in significant different prognosis for the two groups (P = 0.014) (not shown).

The associations between patient age, sex, stage, grade, tumor size, treatment, concomitant CIS, tumor type and Egr-1 protein expression with progression to T2-T4 invasive tumors were tested by univariate Cox regression analysis (table 2). In the univariate analysis Egr-1, patient age, tumor type, tumor stage and tumor grade showed a significant association with progression to T2-T4. Risk factors that were significant (P < 0.01) in the univariate analysis were included in the multivariate analysis. However in the multivariate analysis, Egr-1 expression levels did not prove to be an independent variable for predicting progression to T2-T4 (table 2).

**Table 2:** Univariate and multivariate COX regression analysis

	Univariate analysis		**Multivariate analysis**^a^	
	
Factor	**Hazard ratio**^b^	P	**Hazard ratio**^b^	P
Egr-1				

Low expression (score 1) versus Egr-1 negative (score 0)	1.59 (1.01-2.50)	0.044	1.41 (0.89-2.24)	0.143

Moderate-high expression (score 2-3) versus Egr-1 negative (score 0)	1.95 (1.11-3.44)	0.021	1.38 (0.76-2.49)	0.285

Age (5 year intervals)	1.19 (1.08-1.31)	0.001	1.11 (0.99-1.25)	0.078

Tumor type (Papillary versus solid/mixed)	2.33 (1.43-3.80)	0.001	1.78 (1.03-3.08)	0.038

Tumor stage (T1 versus Ta)	3.01 (2.07-4.38)	<0.001	0.42 (0.16-1.10)	0.078

Tumor grade (High grade versus PUNLMP+low grade)	3.42 (2.34-4.98)	<0.001	6.12 (2.27-16.55)	0.000

Sex	0.99 (0.63-1.56)	0.981		

Size (> 3 cm versus < 3 cm)	1.02 (0.65-1.60)	0.941		

Bacillus Calmette-Guerin (BCG) treatment	0.55 (0.32-0.93)	0.026		

Concomitant CIS	1.02 (0.65-1.60)	0.941		

^a^Only risk factors that were significant (P < 0.01) in the univariate analysis was included in the multivariate analysis.

^b^Number in parentheses, 95% confidence intervals.

### Egr-1 expression localization and intensity of immunolabelling

In some cases, tumor cells with nuclear Egr-1 immunolabelling were found to localize predominantly at the tumor front, i.e. at the border between the bladder tumor and the normal bladder stromal cells (figure 1D). The apparent preponderance of Egr-1 labelling at the tumor front was not found in all specimens. In some cases, tumor cells with nuclear Egr-1 immunolabelling were also found relatively evenly distributed throughout the tumor tissue as illustrated in figure 1 A-C. No statistically significant difference was found in the intensity of Egr-1 nuclear immunolabeling between the Ta tumors and T1 tumors (P = 0.124, not shown).

### Egr-1 expression in non-tumorigenic bladder tissue

The urothelium in three bladder specimens from non-tumorigenic human bladder tissue were examined for Egr-1 immunolabelling. No Egr-1 positive nuclei were found in the urothelium (not shown).

## Discussion

A role for Egr-1 in human bladder cancer progression would be expected from the fact that Egr-1 regulates heparanase and HYAL-1 hyaluronidase expression in human bladder cancer cells [8,9]. In bladder cancer cells upregulation of heparanase expression is shown to occur in a stepwise manner where hypomethylation of the heparanase gene promoter facilitates Egr-1 binding, which is shown to directly regulate the heparanase expression [8]. Heparanase plays a critical role in the degradation of the extracellular matrix and the basement membranes and overexpression is correlated with increased metastatic potential and thereby worse prognosis [21]. Heparanase expression has been found upregulated in gastric, pancreatic, melanoma, prostate and bladder cancers [21-25].

Also the HYAL-1 hyaluronidase expression in bladder cancer has been suggested to be regulated by binding of Egr-1 to the methylated promoter of HYAL-1 hyaluronidase [9].

Hyaluronidase is a highly sensitive and specific marker for high-grade bladder cancer and elevated levels have also been shown in prostate, head, neck and breast cancers and in malignant glioma [9,26-29]. Hyaluronidases degrades hyaluronic acid, which also serves as a tumor marker and promotes metastasis [4,6,29,30].

While heparanase expression was not found in normal bladder tissue, low HYAL-1 hyaluronidase expression was found in normal bladder tissue compared to tumor tissue [8,9]. In the present study, we did not find Egr-1 expression in the three normal human bladder specimens examined (not shown), further supporting a possible role for Egr-1 in human bladder carcinogenesis. Likewise, we previously found that normal rat bladder urothelium expresses no or low levels of Egr-1 [11,19] and Egerod et al., 2009, submitted.

Egr-1 has been shown to regulate the transformation to invasive carcinoma in prostate cancer tumorigenesis, where Egr-1 deficient mice display impaired tumorigenesis [14]. Also inhibition of Egr-1 by antisense oligonucleotides reversed transformation of prostate cancer cells in vitro and in vivo [31].

Because bladder and prostate cancer co-occurs in some patients, it has been suggested that these 2 cancer forms share a common molecular mechanism [32]. The mechanism behind Egr-1 expression in both bladder and prostate cancer are unexplored, but potent inducers of Egr-1 could be stimuli present in tumors *in vivo *such as hypoxia, growth factors and inflammation. One of the clinical implications of our study is that it indirectly supports that the molecular mechanisms underlying bladder and prostate cancer may be similar. Another implication of our study is that it opens new avenues for determining the molecular mechanisms underlying bladder cancer. By performing chromatin immunoprecipitation experiments with Egr-1 antibodies, it may be feasible to determine genes regulated by Egr-1 in invasive human bladder tumor specimens. This would have long-term clinical implications for diagnosis as well as treatment of bladder cancer.

Our study strengthens the role of Egr-1 as a mediator of transformation to invasive bladder cancers. In the univariate analysis Egr-1 was significantly associated with progression to T2-T4; however this was not the case in the multivariante analysis. Therefore Egr-1 cannot be considered an independent marker for progression to T2-T4. Nevertheless, in our study, Egr-1 was still found associated with tumor stage, tumor grade and progression to invasive tumors. Furthermore, we know from previous gene expression studies that Egr-1 is over expressed in muscle invasive tumors. This also emphasizes that Egr-1 may play a role in tumor progression.

CIS is often associated with a high risk of disease progression. Unfortunately, this study included very few CIS lesions, and consequently further work is needed to examine to what extent Egr-1 is expressed in CIS lesions.

## Conclusion

Our study supports a role of Egr-1 in the early steps of human bladder carcinogenesis, with progression from non-muscle invasive tumors to muscle invasive cancers, and emphasizes the need for more comprehensive studies to explore the involvement of Egr-1 in human bladder cancer.

## Competing interests

The authors declare that they have no competing interests.

## Authors' contributions

FLE carried out the immunohistochemical analyses, performed data analysis and part of the statistical analyses, interpretation of data, and drafted the manuscript. AB scored the tissue microarray. NF constructed the tissue microarray and collected follow-up information for all patients in collaboration with LD, MB and TFØ. MBO and NB participated in the conception and design of the study, and critically revised the manuscript. LD performed data analysis and part of the statistical analyses, interpretation of data and critically revised the manuscript. All authors read and approved the final manuscript.

## Pre-publication history

The pre-publication history for this paper can be accessed here:

http://www.biomedcentral.com/1471-2407/9/385/prepub
